# Variations in immune parameters with age in a wild rodent population and links with survival

**DOI:** 10.1002/ece3.9094

**Published:** 2022-07-11

**Authors:** Coraline Bichet, Corinne Régis, Emmanuelle Gilot‐Fromont, Aurélie Cohas

**Affiliations:** ^1^ Centre d'Etudes Biologiques de Chizé CNRS‐La Rochelle Université Villiers‐en‐Bois France; ^2^ Institut für Vogelforschung "Vogelwarte Helgoland" (Institute of Avian Research) Wilhelmshaven Germany; ^3^ UMR‐CNRS 5558, Laboratoire Biométrie et Biologie Évolutive Université Claude Bernard Lyon 1 Villeurbanne France; ^4^ Université de Lyon, VetAgro Sup Marcy‐l'Etoile France; ^5^ Institut Universitaire de France (IUF) Paris France

**Keywords:** aging, immune cells, immune remodeling, immunosenescence, mammal, *Marmota marmota*, survival

## Abstract

Recent findings suggest that immune functions do not unidirectionally deteriorate with age but that a potentially adaptive remodeling, where functions of the immune system get downregulated while others get upregulated with age could also occur. Scarce in wild populations, longitudinal studies are yet necessary to properly understand the patterns and consequences of age variations of the immune system in the wild. Meanwhile, it is challenging to understand if the observed variations in immune parameters with age are due to changes at the within‐individual level or to selective (dis)appearance of individuals with peculiar immune phenotypes. Thanks to a long‐term and longitudinal monitoring of a wild Alpine marmot population, we aimed to understand within‐ and between‐individual variation in the immune phenotype with age, in order to improve our knowledge about the occurrence and the evolutionary consequences of such age variations in the wild. To do so, we recorded the age‐specific leukocyte concentration and leukocyte profile in repeatedly sampled dominant individuals. We then tested whether the potential changes with age were attributable to within‐individual variations and/or selective (dis)appearance. Finally, we investigated if the leukocyte concentration and profiles were correlated to the probability of death at a given age. The leukocyte concentration was stable with age, but the relative number of lymphocytes decreased, while the relative number of neutrophils increased, over the course of an individual's life. Moreover, between individuals of the same age, individuals with fewer lymphocytes but more neutrophils were more likely to die. Therefore, selective disappearance seems to play a role in the age variations of the immune parameters in this population. Further investigations linking age variations in immune phenotype to individual fitness are needed to understand whether remodeling of the immune system with age could or could not be adaptive.

## Graphical abstract

Recent findings suggest that immune functions do not unidirectionally deteriorate with age but that a potentially adaptive remodeling. Scarce in wild populations, longitudinal studies are yet necessary to properly understand the patterns and consequences of age variations of the immune system in the wild. In the meanwhile, it is challenging to understand if the observed variations in immune parameters with age are due to changes at the within‐individual level or to selective (dis)appearance of individuals with peculiar immune phenotypes. Thanks to a long‐term and longitudinal monitoring of a wild Alpine marmot population, we aimed to understand within‐ and between‐individual variation in the immune phenotype with age, in order to improve our knowledge about the occurrence and the evolutionary consequences of such age variations in the wild. We found that the leukocyte concentration was stable with age, but the relative number of lymphocytes decreased, while the relative number of neutrophils increased, over the course of an individual's life. Moreover, between individuals of the same age, individuals with fewer lymphocytes but more neutrophils were more likely to die.

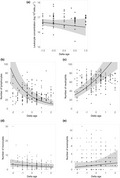

## 1 INTRODUCTION

The immune system is of primary importance to control diseases throughout an individual's life, and therefore crucial to its fitness. In vertebrates, the immune system involves different immune functions which are classically divided into innate and adaptive components (Hoebe et al., 2004); in close interaction with each other (Iwasaki & Medzhitov, 2010, 2015). The innate immune functions are the first defense against pathogens, involving phagocytic cells (e.g., neutrophils, macrophages, and dendritic cells) and molecules, also able to activate other components of the immune system (Akira et al., 2006; Mantovani et al., 2011; Nathan, 2006; Vivier et al., 2011). The adaptive immune functions comprise a cell‐mediated immune response, with the stimulation of T lymphocytes, and a humoral immune response, controlled by activated B lymphocytes that can produce immunoglobulins against specific antigens (Iwasaki & Medzhitov, 2010; Mantovani et al., 2011; Vivier et al., 2011).

Mounting an immune response carries costs (Graham et al., 2005; Lochmiller & Deerenberg, 2000; Maizels & Nussey, 2013) and trade‐offs with other life history traits are likely to emerge (Eraud et al., 2009; Graham et al., 2010; Hanssen et al., 2004; Lemaitre et al., 2015; Viney et al., 2005). Therefore, immunity is likely to change during an individual's life. Changes of immunity with age have been mainly studied in humans and laboratory animals (Bektas et al., 2017; Frasca et al., 2005; Gayoso et al., 2011; Larbi et al., 2008; Noreen et al., 2011; Solana et al., 2012), with the general pattern being a decline in adaptive immunity with age, while innate immunity remains unchanged and inflammatory markers increase (Bauer & De la Fuente, 2016; Franceschi et al., 2007; Franceschi, Bonafe, & Valensin, 2000; Franceschi, Bonafe, Valensin, Olivieri, et al., 2000; Frasca et al., 2011; Panda et al., 2009; Shaw et al., 2013; Simon et al., 2015). In non‐model organisms, a recent review found similar trends (Peters et al., 2019).

Some studies indicate that the decrease in the immune functions with age could impair survival (e.g., Froy et al., 2019; Hanssen et al., 2004; Schneeberger et al., 2014). However, others suggest that variations in immune functions, characterized by changes in the proportion of the different cells involved in the immune response, could be adaptive (i.e., *immune remodeling*) and could fit with the different immune challenges faced throughout life (Fulop et al., 2018; Mueller et al., 2013; Nikolich‐Zugich, 2018). This decrease in the immune functions with age could even be a combination of both fitness impairment and adaptive remodeling (Fulop et al., 2020).

Because the immune system is complex, involving many cell types and pathways, its characterization in non‐model organisms is challenging, thus limiting the study of age‐related variation of immunity in free‐ranging animals and our understanding about the evolutionary consequences of such variations (Boughton et al., 2011; Demas et al., 2011). Nevertheless, cross‐sectional studies investigated the variations in the immune function with age (for instance in mammals: Abolins et al., 2018; Cheynel et al., 2017; Nussey et al., 2012; birds: Hill et al., 2016; Lecomte et al., 2010; Palacios et al., 2007; Saino et al., 2003; Vermeulen et al., 2017; reptiles: Massot et al., 2011; Ujvari & Madsen, 2011; Zimmerman et al., 2013; see Peters et al., 2019 for a review) and seem to confirm the pattern observed in humans and laboratory animals (see above). However, these studies cannot disentangle whether the observed variations arise from within‐individual changes or from processes like selective disappearance, which supposedly eliminate individuals with poor (or inappropriate) immune defenses from the population (van de Pol & Verhulst, 2006; van de Pol & Wright, 2009). Longitudinal studies investigating variations in immune functions with age exist, but are still very limited (to the best of our knowledge, seven studies: Beirne et al., 2016; Bichet et al., 2022; Froy et al., 2019; Graham et al., 2010; Roast et al., 2022; Schneeberger et al., 2014; Vermeulen et al., 2017). Therefore, we are far from understanding how proximate mechanisms, like immunity, could explain (even partly) processes such as aging (Bouwhuis & Vedder, 2017; Lemaitre et al., 2013; Peters et al., 2019).

In the present study, we recorded the age‐specific leukocyte concentration and profile in 52 dominant individuals (i.e., fully grown and reproductive individuals) repeatedly sampled between 2011 and 2015 (for a total of 169 measurements) from a wild and long‐term studied (1992–2018) population of Alpine marmots. We first tested whether leukocyte concentration and profile changed with age in individuals (i.e., within‐individual level). We then investigated if changes in these immune parameters could also be explained by selective (dis)appearance of individuals (i.e., among‐individual level) with particular immune parameters, influencing their risk of death (survival analysis). Based on the previous studies, we expected the relative number of lymphocytes (mainly involved in acquired immunity) to decrease with age, while the relative numbers of other leukocytes (neutrophils, monocytes, eosinophils; mainly involved in innate immunity) to increase with age, at the within‐individual level. We further expect changes in both leukocyte concentration and leukocyte profile to compromise individual age‐specific survival.

## 2 MATERIAL AND METHODS

### 2.1 Studied species

Alpine marmots are territorial, socially monogamous, and cooperatively breeding ground‐dwelling squirrels (Allainé, 2000). They live in families of two to 16 individuals composed of a dominant pair monopolizing reproduction (Arnold & Dittami, 1997; Cohas et al., 2006; Hacklander et al., 2003), sexually mature (≥2 years) subordinates of both sexes, yearlings, and pups of the year (Allainé, 2000). At sexual maturity, subordinates may keep their status, attempt to reach dominance in their natal groups, or disperse to gain dominance in another territory (Lardy et al., 2012). Once an individual reaches dominance, it cannot reverse to subordinate status. Dominance is established for several years and lasts until the dominant individual is evicted or dies (Lardy et al., 2011). During the 23 years of study, only three males and one female lost their dominant status but established dominance in another territory (Lardy et al., 2011).

### 2.2 Field methods

As part of a long‐term study at La Grande Sassière Nature Reserve (2340 m a.s.l., French Alps, 45°29′N, 65°90′E; see Cohas et al., 2008 for details), we captured marmots annually, from mid‐April to mid‐July using live traps placed close to the main burrows to assign trapped individuals to their family. Individuals were marked with a transponder and a numbered ear‐tag, combined with a colored plastic ear‐tag for dominant individuals. At each capture, individuals were tranquilized by an intramuscular injection of Zolétil 100 (0.1 ml.kg^−1^), sexed, aged, weighed, and their social status was determined (large scrotum for dominant males and prominent teats for dominant females, and characteristics of each sex all year round independently of reproduction). Social status was further confirmed by observations of scent‐marking behavior and territorial defense that are characteristics of dominants. The exact age was determined for the individuals born on the study site. For dominant immigrants (five individuals), we assigned the age of three when they first reproduce, as marmots disperse at 2 years old and never reproduce before 3 years old. To determine individual fates, capture histories were combined with intensive observations (each family being observed on average 1 h per day for a minimum of 30 h per year, for details see Cohas et al., 2008). In our population, the maximum lifespan observed over the 30 years of the study is 16 years, and the generation time is about 5 years (Devillard, unpublished data). At each capture, a blood sample (2 ml.kg^−1^ up to 5 ml per individual representing less than 5% of the total volume of blood) was taken from the saphenous vein within 30 min after capture.

To ensure that all the individuals included in this study were in a comparable social status, and all fully grown and reproductive individuals, we restricted the subsequent analysis to the sole dominant marmots, (47 born on the study site and 5 immigrants, removing immigrants did not change qualitatively the results, nor their interpretations). Therefore, our sample is composed of a non‐random subset of individuals, able to survive, and to reach dominance.

### 2.3 Leukocyte concentration

A 20 μl blood‐filled capillary was released in 1 ml of a kit solution (LEUKO‐TIC “blue”, Bioanalytic). This solution allows a microscopic count of leukocytes after the lysis of the erythrocytes and the fixation of the leukocyte nucleus stained in light blue. The counts were done at 1000× enlargement using a Malassez counting chamber by a single observer (C.R.). Only the leukocytes entirely located inside the four 1 mm^2^ corner squares (total volume of 4 × 1 mm^2^ × 0.2 mm = 0.8 μl) were counted. All determinations of leukocyte concentration were done within 24 h after blood collection.

Between 2013 and 2015, the leukocyte concentration was determined for 79 samples from 34 dominant individuals sampled between two and three times. Twenty‐three individuals had two samples in different years and 11 individuals had three. In this dataset, the age of the individuals varied from 3 to 12 years with an average age of 6.2 years (Figure S1A).

### 2.4 Leukocyte profile

Immediately upon blood collection, a drop of blood was smeared onto a slide, later stained with Giemsa stains using an aerospray (Aerospray Hematology Slide/Cytocentrifuge 7150; Wescor). Neutrophils, lymphocytes, monocytes, eosinophils, and basophils were counted (observer: C.R.) for up to 100 leukocytes, at 1000× enlargement, according to Hawkey and Dennett's criteria (Hawkey & Dennett, 1989).

In mammals, lymphocytes and neutrophils make up the majority (80%) of the leukocytes (Jain, 1993). Lymphocytes play a central role in adaptive immunity: they are involved in immunoglobulin (antibodies) production, in the modulation of immune defense, and in the production of memory cells (Jain, 1993; Roitt et al., 2001). Neutrophils are involved in the innate immune response as the primary phagocytic leukocytes, and circulating phagocytes proliferate in response to infections, inflammation, and stress (Jain, 1993). Monocytes are long‐lived phagocytic cells associated with innate defenses against infections and bacteria (Roitt et al., 2001). They differentiate into macrophages in tissues and they are also involved in antigen presentation and cytokine production. Eosinophils play a role in the inflammation process and are associated with defense against internal parasites (Jain, 1993). Basophils, which are rare, play a key role against macroparasites and are also involved in the inflammation process (Karasuyama et al., 2011). The relative number of basophils was low for nearly all individuals (min = 0, max = 49, median = 0) and therefore excluded in the subsequent analyses.

Between 2011 and 2015, the leukocyte profiles were determined for 169 blood smears from 52 dominant individuals sampled between two and five times. Eighteen individuals had two samples in different years, 12 had three, 13 had four, and 9 individuals had five. Thirty‐three of them were both measured for leukocyte concentration and leukocyte counts (which corresponds to 75 measurements). In this dataset, the age of the individuals varied from 3 to 12 years with an average age of 5.8 years (Figure S1B).

### 2.5 Statistical analyses

All statistical analyses were performed with R 3.6.1 (R Core Team, 2014).

#### 2.5.1 Immune phenotype and variation with age

##### Within‐individual variation in immune phenotype with age

To test whether the leukocyte concentration and profiles varied with the age of an individual, we used the leukocyte concentration (log‐transformed) as a dependent variable in a Linear Mixed Model (LMM) and the relative numbers of lymphocytes, neutrophils, monocytes, and eosinophils as dependent variables in four Generalized Linear Mixed Models (GLMMs) with a Poisson distribution (appropriate for the observed distribution of count data). The age was partitioned into “average age” and “delta age” components; where average age represents the among‐individual age effect and delta age the within‐individual age effect (van de Pol & Wright, 2009). The average age was calculated as the average of all ages at which an individual's leukocyte concentration or profile was measured. The delta age corresponds to the difference between the individual age at measurement and its average age (i.e., delta age = age − average age). To investigate the potential non‐linear within‐individual variations of the leukocyte concentration and profile with age, we followed equation (3) recommended by Fay et al. (2022). This equation proposes to model a within‐individual quadratic effect of age as age^2^ − (average age)^2^.

Body mass at capture, capture date, year of capture, and the interaction between capture date and year of capture were further included as fixed effects. Because individuals were sampled several times over the years, we included individual's identities as random intercepts. Our models also included the interaction between “average age” and sex as well as the interactions between sex and both the linear and quadratic effect of “delta age” to test for sex differences in the leukocyte variation with age.

We also conducted the same models as above, but using a subset of our data for which we have both the leukocyte concentration and the relative number of each leukocyte type for the same individuals in the same year (*n* = 75 from 33 individuals). These analyses provided comparable results than the analyses using the whole dataset and are presented in Table S2.

##### Among‐individual variation in immune phenotype with age

Models including average and delta age cannot properly investigate the occurrence of selective appearance and disappearance of peculiar individual phenotypes from the population (Fay et al., 2022; van de Pol & Verhulst, 2006). For this purpose, we replaced the “average age” and “delta age” (linear and quadratic) effects in the previously selected within‐individual models by the actual age, the “age at access to dominance”, to assess the selective appearance, and the “age at last observation”, to assess selective disappearance (Fay et al., 2022; van de Pol & Verhulst, 2006).

The functions “lmer” and “glmer” in the package “lme4” (Bates et al., 2015) were used to fit the models (Bolker et al., 2009). Final models were selected using a backward elimination procedure. We measured zero inflation and variance inflation factors in all our models using the R package “performance” (Lüdecke et al., 2020). For all models, we checked a posteriori distribution of the residuals to assess the fit of the models to the observed data. Since we observed moderate overdispersion (all dispersion ratios <2.58) in some of our models (models for lymphocytes and neutrophils), we estimated all models' parameters using a Bayesian approach. From the final models, we used the “sim” function from the R‐package “arm” to simulate values from the posterior distributions of the model parameters (Gelman & Su, 2020). The 95% credible intervals (CI) around the mean were obtained after 5000 effective simulations. Assessment of statistical support was obtained from the posterior distribution of each parameter. We considered a fixed effect to be important if zero was not included within the 95% CI.

#### 2.5.2 Immune phenotype and survival probability

We tested whether the mortality risk depended on leukocyte characteristics with mixed effects Cox right‐censored regression models (Nenko et al., 2018; Ripatti & Palmgren, 2000; Therneau et al., 2003). These models included leukocyte concentration or profiles as time‐dependent covariates and survival as a response variable using the “coxme” function in the “coxme” R package (Therneau, 2018). The age at first sampling and the sex were also included as fixed effects. Individual identity and year of birth were added as random effects to take into account repeated measurements and cohort effects (Table 2). The data were encoded with a zero as the starting point for all individuals and with the years to death, to the end of the study, or to the next capture (for individuals with repeated data) as right‐censor stop points (Therneau, 2018). For the repeated data, the next interval started with the end of the previous interval. A “1” was assigned to the event variable, if the individual died during the interval. We assumed that an individual died if it was neither captured nor observed the following spring (monitored until 2018). A hazard ratio higher than one indicates that the corresponding explanatory variable is associated with a higher mortality risk. All individuals were followed until death (*n* = 27 for leukocyte concentration and *n* = 43 for leukocyte counts) or still alive in 2018 (*n* = 4 for leukocyte concentration and *n* = 6 for leukocyte counts). Three individuals were excluded from this analysis because their fate (alive or dead) was uncertain, due to a capture permit forbidding the monitoring of their families in 2017 and 2018.

## 3 RESULTS

The relative number of lymphocytes and neutrophils, as well as the relative number of neutrophils and monocytes, were negatively correlated, while the relative number of monocytes and eosinophils were positively correlated (Table S1).

### 3.1 Immune phenotype and variation with age

**FIGURE 1:**
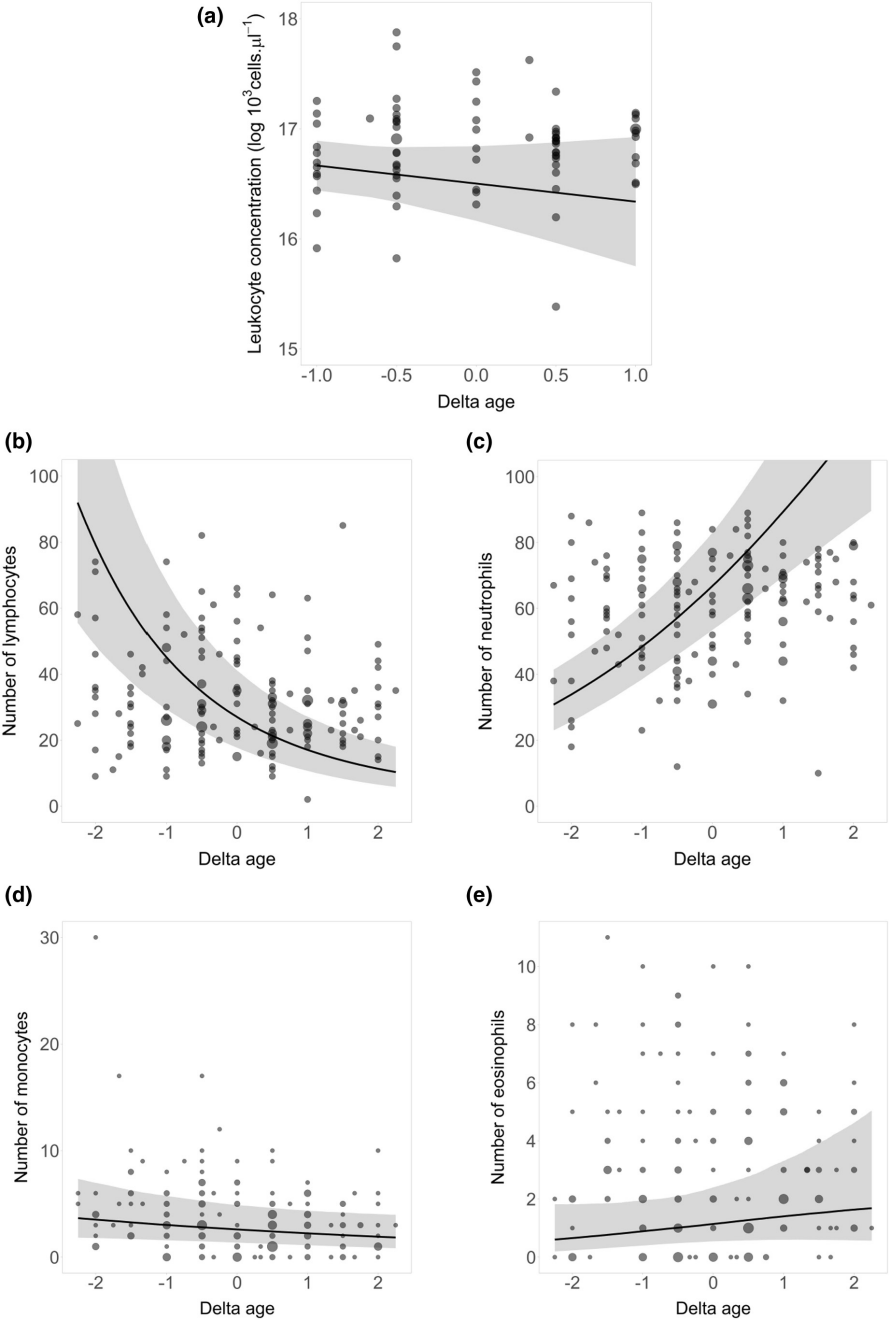
Within‐individual age variations (delta age) of (a) leukocyte concentration and profiles for (b) the relative number of lymphocytes, (c) neutrophils, (d) monocytes, and (e) eosinophils. Dots represent the raw data with size proportional to the sample size. Black solid lines represent model predictions with their 95% prediction interval (gray areas). Model predictions and their intervals were obtained over all samples for (a) and by fixing the continuous variables to their mean over all samples and the sex to female, the year to 2013, and the individual random effect to a given individual for (b–e). The delta age effect was significant for (b) the relative number of lymphocytes, (c) neutrophils, and (e) eosinophils

Over the course of an individual's life, the number of lymphocytes decreased with age (quadratic delta age: *β* = .37, 95% CI = 0.26, 0.47; delta age: *β* = −.56, 95% CI = −0.71, −0.39; Table 1; Figure 1b). Conversely, the number of neutrophils (quadratic delta age: *β* = −.25, 95% CI = −0.32, −0.18; delta age: *β* = .35, 95% CI = 0.25, 0.45; Table 1; Figure 1c) and eosinophils (quadratic delta age: *β* = −.36, 95% CI = −0.711, −0.019; delta age: *β* = .26, 95% CI = −0.62, 0.67; Table 1; Figure 1e) increased while an individual aged.

**TABLE 1:** Parameter estimates and credible intervals at 95% (CI) for the selected models testing whether within‐individual variation in leukocyte concentration or relative number of each type of leukocytes was explained by age. Parameters were obtained from the minimal adequate models. Significant effects (CI which do not overlap zero) are in bold. “—” means a parameter not retained in the model

Dependent variable	Leukocyte concentration^a^		Number of lymphocytes^b^		Number of neutrophils^b^		Number of monocytes^b^		Number of eosinophils^b^	
Fixed effects	Estimate	95% CI	Estimate	95% CI	Estimate	95% CI	Estimate	95% CI	Estimate	95% CI
Intercept	**16.51**	**16.17, 16.86**	**3.27**	**3.00, 3.54**	**4.2**	**4.05, 4.35**	**1.27**	**0.90, 1.65**	**0.61**	**0.15, 1.09**
Average age	−0.06	−0.16, 0.05	−0.01	−0.10, 0.08	0.01	−0.04, 0.06	−0.1	−0.21, 0.02	0.09	−0.05, 0.23
Delta age	−0.11	−0.31, 0.09	**−0.55**	**−0.71, −0.39**	**0.35**	**0.25, 0.45**	−0.17	−0.35, 0.01	0.26	−0.62, 0.67
Quadratic delta age	—	—	**0.37**	**0.26, 0.47**	**−0.25**	**−0.32, −0.18**	—	—	**−0.37**	**−0.71, −0.02**
Sex (male)	−0.09	−0.30, 0.13	**−0.22**	**−0.41, −0.04**	0.07	−0.03, 0.17	0.08	−0.15, 0.31	0.01	−0.28, 0.30
Body mass	−0.05	−0.17, 0.08	**0.14**	**0.08, 0.20**	**−0.08**	**−0.12, −0.04**	**0.21**	**0.09, 0.33**	**0.23**	**0.08, 0.37**
Date	0.06	−0.08, 0.20	**0.09**	**0.03, 0.15**	−0.01	−0.04, 0.03	0.08	−0.06, 0.23	0.07	−0.07, 0.21
Year (2012)			0.09	−0.07, 0.26	−0.08	−0.19, 0.03	0.16	−0.16, 0.48	**0.72**	**0.33, 1.11**
Year (2013)			**0.45**	**0.18, 0.72**	**−0.2**	**−0.35, −0.05**	−0.34	−0.83, 0.14	−0.11	−0.67, 0.45
Year (2014)	**0.44**	**0.10, 0.78**	0.32	−0.03, 0.66	−0.16	−0.34, 0.02	−0.09	−0.63, 0.32	0.25	−0.34, 0.83
Year (2015)	0.49	−0.06, 1.04	0.35	−0.11, 0.80	−0.19	−0.34, 0.02	−0.23	−0.75, 0.47	0.62	−0.12, 1.36
Date: year 2012			**−0.27**	**−0.36, −0.17**	—	—	0.11	−0.11, 0.33	—	—
Date: year 2013			**−0.2**	**−0.31, −0.10**	—	—	**−0.51**	**−0.88, −0.14**	—	—
Date: year 2014	—	—	−0.07	−0.20, −0.06	—	—	−0.09	−0.42, 0.23	—	—
Date: year 2015	—	—	−0.07	−0.19, 0.06	—	—	−0.23	−0.61, 0.15	—	—
Random ID (variance)	**0.03**	**0.02, 0.04**	**0.1**	**0.08, 0.13**	**0.03**	**0.02, 0.03**	**0.07**	**0.05, 0.11**	**0.12**	**0.09, 0.18**

^a^ Between 2013 and 2015, the leukocyte concentration was determined for 79 samples from 34 individuals sampled between two and three times. Twenty‐three individuals had two samples in different years and 11 individuals had three.

^b^ Between 2011 and 2015, the leukocyte counts were determined for 169 blood smears from 52 individuals sampled between two and five times. Eighteen individuals had two samples in different years, 12 had three, 13 had four, and 9 individuals had five. Thirty‐three individuals were both measured for leukocyte concentration and leukocyte counts (which corresponds to 75 measurements).

The age at access to dominance did not affect either lymphocytes' or neutrophils' relative numbers (Table 2). However, among individuals, older ages at last observation were associated with higher numbers of lymphocytes (*β* = .27, 95% CI = 0.15, 0.40) and lower numbers of neutrophils (*β* = −.15, 95% CI = −0.21, −0.08) were. This indicates no selective appearance but selective disappearance for individuals with low number of lymphocytes and high number of neutrophils.

**TABLE 2:** Parameter estimates and credible intervals at 95% (CI) for the selected models testing whether variation in leukocyte concentration or relative number of each type of leukocytes was explained by age, age at access to dominance (AAD, i.e., selective appearance), and age at last observation (ALO, i.e., selective disappearance). Parameters were obtained from the minimal adequate models. Significant effects (CI which do not overlap zero) are in bold. “—” means a parameter not retained in the model

Dependent variable	Leukocyte concentration^a^		Number of lymphocytes^b^		Number of neutrophils^b^		Number of monocytes^b^		Number of eosinophils^b^	
Fixed effects	Estimate	95% CI	Estimate	95% CI	Estimate	95% CI	Estimate	95% CI	Estimate	95% CI
Intercept	16.7	16.47, 16.92	3.51	3.35, 3.67	4.03	3.94, 4.13	1.34	1.08, 1.60	0.76	0.43, 1.09
Age	0.09	−0.11, 0.30	−0.28	−0.39, −0.17	0.14	0.08, 0.20	−0.32	−0.49, −0.14	−0.01	−0.22, 0.19
AAD	−0.12	−0.25, 0.00	−0.01	−0.11, 0.10	0.02	−0.03, 0.07	0.04	−0.10, 0.19	−0.06	−0.22, 0.11
ALO	−0.09	−0.28, 0.09	**0.27**	**0.15, 0.39**	**−0.15**	**−0.21, −0.08**	0.16	−0.01, 0.33	0.13	−0.08, 0.35
Sex (male)	−0.09	−0.29, 0.12	**−0.3**	**−0.47, −0.12**	**0.12**	**0.04, 0.22**	0.04	−0.19, 0.28	−0.06	−0.35, 0.23
Body mass	−0.05	−0.17, 0.07	**0.15**	**0.09, 0.21**	**−0.09**	**−0.13, −0.05**	**0.23**	**0.11, 0.35**	**0.24**	**0.10, 0.38**
Date	0.06	−0.08, 0.20	**0.13**	**0.07, 0.19**	**−0.08**	**−0.12, −0.03**	0.07	−0.07, 0.21	0.05	−0.09, 0.18
Year (2012)			−0.03	−016, 0.10	0.01	−0.09, 0.10	0.17	−0.12, 0.45	**0.7**	**0.35, 1.05**
Year (2013)			**0.26**	**0.09, 0.44**	−0.08	−0.20, 0.04	−0.34	−0.75, 0.07	−0.2	−0.67, 0.26
Year (2014)	**0.28**	**0.02, 0.54**	0.09	−0.08, 0.27	0	−0.11, 0.11	−0.2	−0.52, 0.12	0.07	−0.32, 0.46
Year (2015)	0.14	−0.14, 0.43	0.08	−0.14, 0.30	0	−0.13, 0.13	−0.2	−0.57, 0.17	0.38	−0.07, 0.82
Date: year 2012			**−0.29**	**−0.39, −0.120**	**0.12**	**0.05, 0.19**	0.11	−0.11, 0.33	—	—
Date: year 2013			**−0.22**	**−0.33, −0.12**	**0.09**	**0.01, 0.17**	**−0.5**	**−0.87, −0.12**	—	—
Date: year 2014	—	—	−0.13	−0.26, −0.00	0.07	−0.02, 0.17	−0.1	−0.43, 0.23	—	—
Date: year 2015	—	—	−0.1	−0.22, 0.02	0.07	−0.02, 0.15	−0.24	−0.62, 0.15	—	—
Random ID (variance)	0.02	0.01, 0.03	**0.08**	**0.07, 0.11**	**0.02**	**0.01, 0.02**	**0.06**	**0.05, 0.09**	**0.12**	**0.09, 0.17**

^a^ Between 2013 and 2015, the leukocyte concentration was determined for 79 samples from 34 individuals sampled between two and three times. Twenty‐three individuals had two samples in different years and 11 individuals had three.

^b^ Between 2011 and 2015, the leukocyte counts were determined for 169 blood smears from 52 individuals sampled between two and five times. Eighteen individuals had two samples in different years, 12 had three, 13 had four, and 9 individuals had five. Thirty‐three individuals were both measured for leukocyte concentration and leukocyte counts (which corresponds to 75 measurements).

The leukocyte concentration (Figure 1a) and the number of monocytes (Figure 1d) did not vary with age (both among‐ and within‐individual, linear or quadratic effects were not significant, Table 1) and were not affected by age at access to dominance nor by age at last observation (Table 2).

We did not find any difference in the age trajectories between males and females (non‐significant interactions between “delta age” and “sex” removed from the final models). We also did not observe sex differences in the average leukocyte concentration and profiles, except that males had a lower relative abundance of lymphocytes than females (*β* = −.22, 95% CI = −0.41, −0.04, Table 1).

### 3.2 Immune phenotype and survival probability

We detected a positive correlation between the leukocyte concentration and the mortality risk at a given age (Table 3). Moreover, we found that the relative number of neutrophils was positively correlated, while the relative number of lymphocytes was negatively correlated, with the mortality risk (Table 3). The relative numbers of monocytes and eosinophils were not correlated with the mortality risk (Table 3). These results are consistent with the results provided by the previous models.

**TABLE 3:** Associations between immune phenotype and the mortality risk. Significant effects are in bold

Time‐dependent covariate	Leukocyte concentration (*N* = 72, *n* = 27 events)			Number of lymphocytes (*N* = 163, *n* = 43 events)			Number of neutrophils (*N* = 163, *n* = 43 events)			Number of monocytes (*N* = 163, *n* = 43 events)			Number of eosinophils (*N* = 163, *n* = 43 events)		
	Hazard ratio ± *SE*	*Z* value	*p*‐value	Hazard ratio ± *SE*	*Z* value	*p*‐value	Hazard ratio ± *SE*	*Z* value	*p*‐value	Hazard ratio ± *SE*	*Z* value	*p*‐value	Hazard ratio ± *SE*	*Z* value	*p*‐value
Leukocyte variable	**1.00 ± 0.00**	**1.97**	**.049**	**0.96 ± 0.01**	**−3.00**	**.003**	**1.03 ± 0.01**	**2.80**	**.005**	0.93 ± 0.07	−1.02	.310	1.04 ± 0.07	0.57	.570
Age at first capture	**1.31 ± 0.10**	**2.68**	**.007**	1.11 ± 0.09	1.21	.230	1.12 ± 0.09	1.30	.190	1.07 ± 0.09	0.78	.440	1.09 ± 0.09	0.89	.370
Sex (male)	0.98 ± 0.40	−0.04	.970	−0.53 ± 0.33	−1.90	.058	0.61 ± 0.33	−1.54	.120	0.72 ± 0.32	−1.02	.310	0.69 ± 0.32	−1.18	.240

## 4 DISCUSSION

The immune parameters (leukocyte concentration and leukocyte profile) measured on the marmots varied with their age. At the within‐individual level, although the leukocyte concentration remains stable over the course of an individual life, the relative number of lymphocytes decreased, while the relative numbers of neutrophils and eosinophils increased with age. These results were consistent when a subset of our data for which we have both the leukocyte concentration and the relative number of each leukocyte type for the same individuals in the same year was used in the analyses (Table S2). These results were also corroborated by the absence of correlation between the leukocyte concentration and any relative numbers of leukocyte types (Table S1).

The decrease in the number of lymphocytes with age is often interpreted as a consequence of the gradual decline over age in the generation, in the thymus, of new naïve T lymphocytes, responsible for generating new input in the immune memory. Such gradual decline is often suggested to lead to a decrease in the efficiency of the acquired immune system (Dowling & Hodgkin, 2009; Hakim & Gress, 2007; Shanley et al., 2009). The observed increase in the relative number of neutrophils does not necessarily mean a higher performance of the innate immune system with age. Indeed, the phagocytic ability of neutrophils could decrease with age (Gomez et al., 2008) and a compensatory mechanism for such a decline in neutrophil performance could lead to an increase in their relative number.

However, a decrease in lymphocytes, together with an increase in neutrophils (Cheynel et al., 2017; Kirk et al., 2010; and in Roast et al., 2022, even if not significant), and more broadly, a decrease in the acquired immune system combined with an increase (or upkeep) in the innate immune system, with age, has been observed in various vertebrate species (Franceschi, Bonafe, & Valensin, 2000; Franceschi, Bonafe, Valensin, Olivieri, et al., 2000; reviewed in Peters et al., 2019). Such modification of the acquired/innate immune balance with age (McDade et al., 2016), called *immune remodeling*, could be interpreted as an increased resource allocation strategy toward the production of cheaper immune components, at the expense of the more costly immune components (Klasing, 2004). Indeed, the acquired immune components are generally thought to be more expensive to maintain (Lee, 2006), and therefore, more prone to decline with age than the innate immune components (Peters et al., 2019). This could reflect that immune parameters are traded‐off with other life history traits, and/or that terminal investment is unlikely to be turned to these parameters since their costs of production would outweigh their future benefit when age‐related mortality becomes imminent. In such a scenario, given the lower probability to encounter new pathogens at old ages, downregulating the acquired immune system would not necessarily be the sign of any malfunction, but could be adaptive (Fulop et al., 2018). Consequently, immune systems should not be considered to undergo unidirectional deterioration with age (i.e., senescence) but would probably be better described by taking into account remodeling and reshaping of the immune functions with age (Fulop et al., 2018).

Apart from the immune remodeling hypothesis exposed above, age‐related changes in the level of environmental stress endured by an individual could also affect the relative numbers of lymphocytes and neutrophils. Indeed, stress hormones such as glucocorticoids stimulate an influx of neutrophils from tissues into the blood; concomitantly, it causes a migration of lymphocytes from the blood circulation to other compartments (Dhabhar, 2002). Thus, a rise of plasma glucocorticoids caused by stress increases the neutrophils to lymphocytes ratio over a time span of hours (Davis et al., 2008; Lopez‐Olvera et al., 2007). Nevertheless, to date, no link has been clearly established between environmental stress, levels of glucocorticoids, and patterns of age variations of lymphocytes and neutrophils (see for instance Roast et al., 2022; Watson et al., 2016).

We observed a lower relative abundance of lymphocytes for marmot males than for females. Various hypotheses such as sex differences in resource allocation strategy, intrasexual competition (Metcalf & Graham, 2018; Sheldon & Verhulst, 1996), or inhibition of the immune system by some steroid hormones were often suggested to induce immune differences between males and females (Gubbels Bupp et al., 2018; Klein & Flanagan, 2016; Taneja, 2018). However, we did not observe sex‐specific differences in the variation of the immune phenotype with age. So far, studies of sex‐specific variation on immune parameters with age remain equivocal: some suggested sex differences (e.g., Bichet et al., 2022; Gubbels Bupp et al., 2018; Tidière et al., 2020; van Lieshout et al., 2020), while others did not (e.g., Brooks & Garratt, 2017; Cheynel et al., 2017; Kelly et al., 2018; Peters et al., 2019). For instance, van Lieshout et al. (2020) found a decrease in the proportion of lymphocytes with age in male badgers (*Meles meles*), but not in females. The authors argued that this result could be explained by the high testosterone levels observed in male badgers, due to their polygynandrous mating system (Buesching et al., 2009), contrary to monogamous species (Sugianto et al., 2019) such as the Alpine marmot (Allainé, 2000; Cohas et al., 2006).

In our study, individuals with proportionally fewer lymphocytes but more neutrophils were more likely to die (Table 3), as also indicated by a significant selective disappearance of individuals with this phenotype (Table 2). Innate cellular response (involving neutrophils) can be costly in terms of energy, as well as autoimmune (Lee, 2006) and inflammatory damages (Franceschi et al., 2018; Goto, 2008). Individuals with neutrophil‐oriented response may be unable to mount an appropriate immune response against challenges encountered at old ages (Froy et al., 2019) and/or may pay an excessive cost to this response and die (Pawelec, 2018). Studies investigating the potential links between age variation in immune phenotype and individual fitness are still scarce and show contrasting results (see also Froy et al., 2019). For instance, in the greater sac‐winged bat (*Saccopteryx bilineata*), the number of leukocytes decreased with age, both within and among individuals, while the immunoglobulin G concentration was higher in older individuals, but did not vary within individuals, and the bacterial killing capacity of the plasma did not vary with age, at both levels (Schneeberger et al., 2014). These variations with age also impacted the short‐term survival probability (Schneeberger et al., 2014). However, in a study on purple‐crowned fairywrens (*Malurus coronatus*), Roast et al. (2020) found no evidence that high levels of innate immune functions impaired short‐term survival, nor any other fitness traits (annual reproduction and dominance acquisition). In our marmot population, more investigations on the link between fitness and immune variations with age are needed to better understand the evolutionary consequences of the within‐individual age variations and the selective disappearance we observed.

More generally, to understand the complexity of age‐related changes in immune functions, as well as their evolutionary causes and consequences, we must not only supplement the existing longitudinal studies focused on age‐related pattern of immune parameters (to the best of our knowledge, seven studies: Beirne et al., 2016; Bichet et al., 2022; Froy et al., 2019; Graham et al., 2010; Roast et al., 2022; Schneeberger et al., 2014; Vermeulen et al., 2017), but also relate the observed patterns to individual fitness. Such studies are crucial to disentangle whether remodeling of the immune system with age could or could not be adaptive.

## AUTHOR CONTRIBUTIONS

**Coraline Bichet:** Conceptualization (equal); formal analysis (lead); writing – original draft (lead); writing – review and editing (lead). **Corinne Régis:** Methodology (lead). **Emmanuelle Gilot‐Fromont:** Conceptualization (supporting); methodology (supporting); writing – review and editing (supporting). **Aurélie Cohas:** Conceptualization (equal); data curation (lead); formal analysis (supporting); funding acquisition (lead); project administration (lead); writing – original draft (lead); writing – review and editing (lead).

## CONFLICT OF INTEREST

The authors declare that they have no conflict of interest.

## Supporting information


Appendix S1

## Data Availability

Data are available from the Dryad Digital Repository: https://doi.org/10.5061/dryad.bvq83bk5d.
