# Supplementary material for: Variations in immune parameters with age in a wild rodent population and links with survival
Source: Ecol Evol. 2022 Jul 11;12(7):e9094. doi: 10.1002/ece3.9094 (PMC9273568; doi:10.1002/ece3.9094)
Supplement: Supplementary file 1 — Appendix S1 [file ECE3-12-e9094-s001.docx]

**Supplemental Information for:**

**Variations in immune parameters with age in a wild rodent population and links with survival**

Coraline Bichet, Corinne Regis, Emmanuelle Gilot-Fromont and Aurélie Cohas

**Table S1:** Pearson coefficients of correlation between the leukocyte concentration and the relative number of lymphocytes, neutrophils, monocytes and eosinophils. Values in brackets represent the 95% confidence intervals. Significant correlations are in bold.

|  | Leucocyte concentration | Number of lymphocytes | Number of neutrophils | Number of monocytes |
| --- | --- | --- | --- | --- |
| Number of lymphocytes | 0.04 [-0.18;0.27]  t = 0.37  n = 75  p = 0.71 | **-** |  |  |
| Number of neutrophils | -0.02 [-0.24;0.21]  t = -0.15  n = 75  p = 0.88 | **-0.93 [-0.90;-0.94]**  **t = -31.34**  **n = 169**  **p < 0.001** | - |  |
| Number of monocytes | -0.11 [-0.33;0.13]  t = -0.94  n = 75  p = 0.35 | 0.08 [-0.07;0.23]  t = 1.05  n = 169  p = 0.30 | **-0.32 [-0.45;-0.18]**  **t = -4.44**  **n = 169**  **p < 0.001** | - |
| Number of eosinophils | -0.03 [-0.26;0.19]  t = -0.29  n = 75  p = 0.77 | -0.12 [-0.27;0.03]  t = -1.57  n = 169  p = 0.12 | -0.11 [-0.26;0.04]  t = -1.44  n = 169  p = 0.15 | **0.23 [0.08;0.36]**  **t = 3.00,**  **n = 169**  **p < 0.01** |


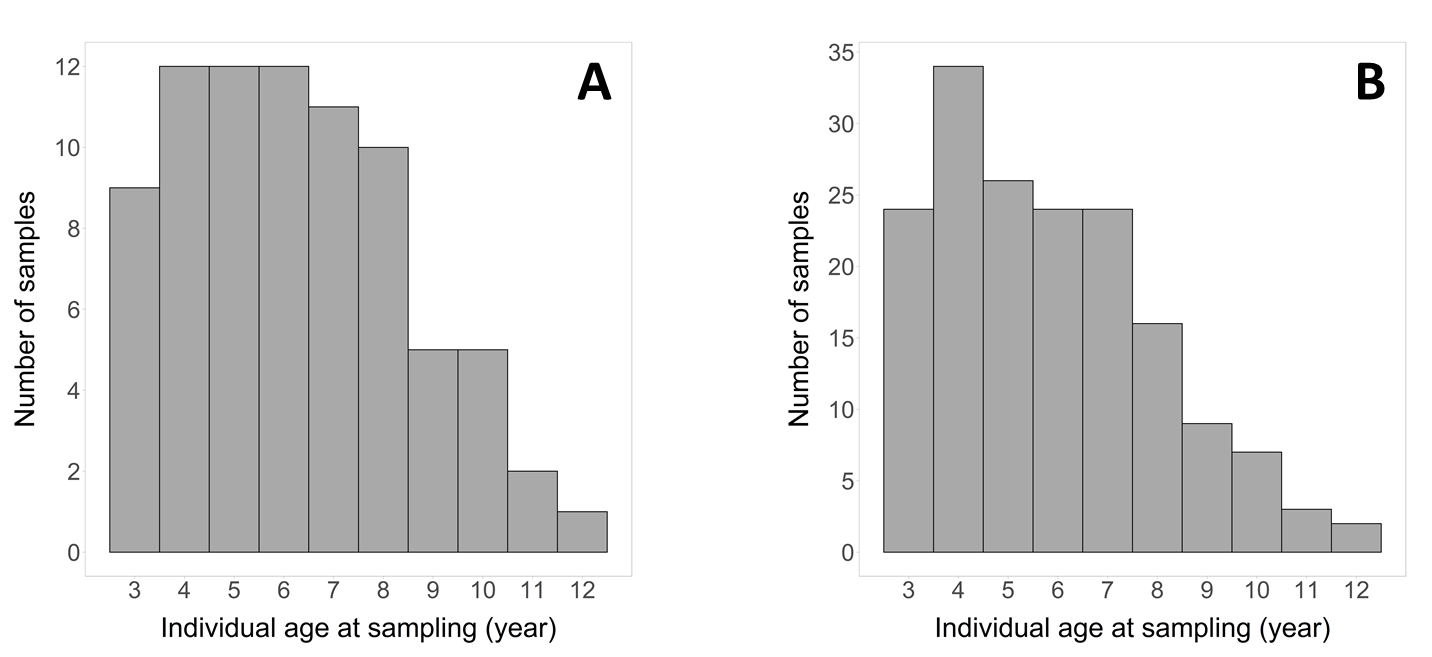


**Figure S1:** Age-distribution at sampling of the marmot individuals included in the study, for (A) leukocyte concentration and (B) leukocyte profile.

**Table S2:** Parameter estimates and credible intervals at 95% (CI) for the selected models testing whether within-individual variation in leukocyte concentration or relative number of each type of leukocytes were explained by age, for a subset of individuals for which we have both leukocyte concentration and the relative number of each type of leukocytes in the same year (n = 75 from 33 individuals). Parameters were obtained from the minimal adequate models. Significant effects (CI which do not overlap zero) are in bold. “-“ means a parameter not retained in the model.

| Dependant variable | **Leukocyte concentration** | | **Number of lymphocytes** | | **Number of neutrophils** | | **Number of monocytes** | | **Number of eosinophils** | |
| --- | --- | --- | --- | --- | --- | --- | --- | --- | --- | --- |
| Fixed effects | Estimate | 95% CI | Estimate | 95% CI | Estimate | 95% CI | Estimate | 95% CI | Estimate | 95% CI |
| Intercept | **16.59** | **16.34, 16.85** | **4.15** | **3.88, 4.42** | **3.60** | **3.41, 3.79** | **1.36** | **0.93, 1.81** | **0.53** | **0.04, 1.03** |
| Average age | -0.00 | -0.15, 0.14 | -0.09 | -0.28, 0.09 | 0.09 | -0.01, 0.20 | -0.11 | -0.40, 0.19 | -0.17 | -0.45, 0.10 |
| Delta age | -0.17 | -0.42, 0.08 | **-0.52** | **-0.86, -0.19** | **0.42** | **0.20, 0.63** | -0.09 | -0.54, 0.37 | 0.21 | -0.21, 0.63 |
| Quadratic delta age | - | - | **0.42** | **0.24, 0.59** | **-0.33** | **-0.46, -0.20** | - | - | - | - |
| Sex (male) | -0.07 | -0.29, 0.14 | **-0.24** | **-0.41, -0.04** | 0.12 | -0.03, 0.27 | -0.05 | -0.47, 0.36 | 0.28 | -0.12, 0.68 |
| Body mass | -0.04 | -0.18, 0.10 | **0.23** | **0.08, 0.20** | **-0.14** | **-0.21, -0.06** | **0.49** | **0.23, 0.74** | 0.22 | -0.03, 0.46 |
| Date | 0.03 | -0.12, 0.18 | -0.02 | -0.49, 0.02 | 0.00 | -0.07, 0.07 | **-0.37** | **-0.65, -0.08** | 0.16 | -0.10, 0.42 |
| Year (2014) | **0.44** | **0.12, 0.75** | **-0.33** | **-0.62, -0.03** | **0.21** | **0.03, 0.39** | -0.29 | -0.84, 0.25 | 0.09 | -0.50, 0.68 |
| Year (2015) | **0.49** | **0.04, 0.94** | -0.34 | -0.86, 0.19 | 0.23 | -0.08, 0.53 | -0.31 | -1.11, 0.51 | 0.19 | -0.61, 0.96 |
|  |  |  |  |  |  |  |  |  |  |  |
| Random ID (variance) | **0.02** | **0.01, 0.04** | **0.11** | **0.09, 0.17** | **0.03** | **0.03, 0.05** | **0.14** | **0.09, 0.22** | **0.10** | **0.06, 0.16** |
